# Fitness of Insect-resistant transgenic rice T1C-19 under four growing conditions combining land use and weed competition

**DOI:** 10.1080/21645698.2021.1914290

**Published:** 2021-04-21

**Authors:** Jianmei Fu, Biao Liu, Laipan Liu, Zhixiang Fang

**Affiliations:** a State Environmental Protection Key Laboratory on Biosafety, Research Center for Biodiversity Conservation and Biosafety, Nanjing Institute of Environmental Sciences, Ministry of Ecology and Environment, Nanjing, China; b Department of Rice Pest, Institute of Plant Protection, Jiangsu Academy of Agricultural Sciences, Nanjing, China; c State Environmental Protection Scientific Observation and Research Station for Ecology and Environment of Wuyi Mountains, Nanjing Institute of Environmental Sciences, Ministry of Ecology and Environment, Nanjing, China; d College of Life Sciences, Nanjing Agricultural University, NanjingChina

**Keywords:** Insect-resistant transgenic rice, uncultivated land, weed competition, expression of exogenous protein, fitness

## Abstract

Transgene escape into natural ecosystems through seed spraying or transgene introgression may potentially cause environmental biosafety problems. In this study, we assessed the environmental risk of insect-resistant transgenic rice entering farmland margins or natural ecosystems adjacent to farmland. Transgenic Cry1C* rice (T1C-19) was used to study the effects of exogenous Cry1C* expression on vegetative and reproductive growth indices under different growing conditions using the following four combined treatments of land use and weeds: farmland and uncultivated land without weeds (F–NW and U–NW, respectively), and farmland and uncultivated land with weeds (F–W and U–W, respectively). The expression of Cry1C* protein under the U–NW, F–W, and U–W conditions was significantly lower than under the control condition, F–NW. Tiller number, biomass, filled grain number, filled grain weight, and other vegetative and reproductive indices were significantly lower in the rice line TIC-19 than in MH63 under F–NW and U–NW conditions, indicating a significant fitness cost. However, under F–W and U–W conditions, vegetative growth indices such as plant height, tiller number, and biomass, as well as reproductive growth indices such as filled grain number per plant, filled grain weight per plant, and seed setting rate in TIC-19 were similar to those in MH63, indicating a long-term coexistence. These results indicate a lower ecological risk of T1C-19 compared to MH63 under F–NW and U–NW, although their long-term coexistence may lead to potential ecological risks under F–W and U–W.

## Introduction

*Bacillus thuringiensis* (*Bt)* insecticidal-protein genes are the most extensively studied, most widely used, and most promising insect resistance-related genes. BT proteins show strong specific toxicity to *Lepidoptera*, and have been widely used commercially in transgenic corn and cotton crops, giving rise to significant economic, environmental, and social benefits.^1^ Currently, rice (*Oryza sativa*) is the most important food crop in the world, and has, therefore, garnered the attention of researchers for the development of insect–resistant transgenic rice, and major breakthroughs have been accomplished.^2–7^ Indeed, in 2006 the insect-resistant Cry1C* transgenic rice line, T1C-19, was successfully developed with high target insect resistance while meeting the technical requirement for commercial production. However, there are concerns regarding its environmental biosafety because T1C-19 rice is likely to be commercially planted on a large scale in the future. These concerns include the ecological risks caused by exogenous gene escape and the associated negative impacts on non–target organisms, target organisms, soil microbial communities and biodiversity, among other hazards.^8–12^ In this present study, we assessed the possible environmental risks caused by the introduction of Bt transgenic rice in farmland margins or natural ecosystems overlapping with surrounding farmland through volunteer plants or gene flow. Our findings will provide important theoretical guidance for predicting the long-term ecological risk caused by large-scale commercial planting of this transgenic rice variety.

Studying the expression of exogenous proteins over the entire life cycle in transgenic rice after escaping into a natural ecosystem is the first step in evaluating the ecological risk posed by the introduction of transgenic crops in natural ecosystems. Such studies can provide a sound theoretical basis for rational layout and scientific management of transgenic rice in the future. In theory, when exogenous genes are expressed normally under natural conditions, it is possible to alter the fitness of the recipient rice plants, including its ability for competition and adaption, further affecting the evolutionary potential of its wild relatives, and thus leading to potential ecological consequences.

Nearly 20 years of studying the fitness of transgenic crops in farmland ecosystems have significantly increased our understanding of the phenomena involved. Research has shown that exogenous *Bt* genes are likely to bring fitness benefits to recipient rice by conferring insect resistance under high target insect pressure in the field or in greenhouses. For example, Tang et al.^5^ found that in the field and under natural, high insect pressure, the total yield of insect-resistant transgenic Cry1C* rice T1C-19 was significantly higher than that of parental line, MH63. Similar results were reported by Jiang et al.,^13^ who found that the yield of T1C-19 was significantly higher (8.4%) than that of MH63 in the field and under high target insect pressure. Conversely, other studies have found that the field performance of transgenic crops is not always better than that of the parental line. In fact, some studies have shown reduced performance of the transgenic line (reduced plant height and root length, fewer grains per panicle, and reduced seed–setting rates), under low or even in the absence of insect pressure,^3,14–17^ indicating the associated fitness costs. However, except for a few studies on crop species other than rice, there are currently no reports on the fitness of insect-resistant transgenic rice after it has entered uncultivated land through volunteer plants or gene flow and is faced with competing weeds in the natural ecosystems adjacent to the farmland where it was cultivated.

Artificial application of selection pressures found in the environment, such as biotic (i.e. weeds) and abiotic stress (i.e. soil) factors, to the controlled experimental conditions can reveal the fitness costs or benefits associated with the ecological risks posed by transgenic crops escaping into the natural ecosystem. This can enhance our understanding of the ecological risks associated with large-scale commercial planting of transgenic crops. In addition, the population of target insects in farmland ecosystems and natural ecosystems may be controlled and maintained at a low level following large-scale commercial planting of transgenic crops in the future.

This greenhouse study simulated the escape of transgenic rice from farmland. We studied the effects under the following treatment conditions: farmland without weed competition (F–NW, control farmland), uncultivated land without weed competition (U–NW, semi-natural), farmland with weed competition (F–W, semi-natural), and uncultivated land with weed competition (U–W, natural), without target insect pressure, (1) to detect the expression of Cry1C* protein in transgenic T1C-19 rice in different tissues during different growth stages; (2) to investigate the vegetative and reproductive growth fitness of T1C-19 rice under different growth conditions; and (3) to analyze possible reasons for fitness changes in T1C-19 rice under different growth conditions. We aimed to provide a theoretical basis for future evaluation of the long-term environmental risks of insect-resistant transgenic rice T1C-19 caused by large-scale commercial planting.

## Materials and Methods

### Rice

Transgenic Cry1C* rice T1C-19 (T1C-19 rice), an important transgenic rice breeding material in China, and parental rice Minghui63 (MH63), a most widely used restorer line in China, were used in the present study and provided by National Key Laboratory of Crop Genetic Improvement, Wuhan, China. The Cry1C* gene for improving the resistance to lepidopteran insects was synthesized on the basis of wild-type *Cry1Ca5* gene of *Bacillus thuringiensis*.^5^

### Land Use Type and Weeds

We simulated four growth conditions with different combinations of two types of land use with or without weed control. Uncultivated land typical yellow topsoil (topsoil, 0–30 cm layer) was collected from a natural uncultivated land with virgin soil in Jiangning District, Nanjing, Jiangsu (31°37ʹ-32°07ʹ N, 118° 28ʹ-119°06ʹ E). The control farmland soil (topsoil, 0–30 cm layer) was collected from a paddy field in Liuhe District, Nanjing, Jiangsu (32°11ʹ-32°27ʹ N, 118°34 ‘-119°03ʹ E). The physicochemical properties of soils were determined (Table 1), and the organic matter, total nitrogen (N), total P, available P, total potassium (K), and the available K, concentrations in the uncultivated land soil with or without weed were significantly lower than the corresponding concentrations in farmland soil with or without weed (*P* < .01). According to the Classification of Early Arable Land in Red and Yellow Soil in the Southern Mountains and Hills in the People’s Republic of China, Agricultural Industry Standard NY/T309-1996, the fertility level of the test uncultivated yellow land soil is 8–9, which is consistent with the experimental requirement of uncultivated land considering poor concentrations of key nutrition elements.

**Table 1.** Physical and chemical properties of the two soils in this study

Physicochemical property of soil	Farmland	Uncultivated land
Organic matter (g/kg)	40.41 ± 0.15**	12.86 ± 1.22
Total nitrogen content (g/kg)	1.83 ± 0.05**	0.72 ± 0.09
Total phosphorus content (g/kg)	1.44 ± 0.06**	0.39 ± 0.02
Total potassium content (g/kg)	12.21 ± 0.14*	10.14 ± 0.08
Available phosphorus content (mg/kg)	160.22 ± 12.3**	11.62 ± 2.16
Available potassium content (mg/kg)	0.38 ± 0.04**	0.12 ± 0.04

Physicochemical properties of soil for * and ** are significantly different from two soils according to *t*-test (*P* < 0.05) and (*P* < 0.01), respectively (n = 3).

Considering high weed cover in wild uncultivated land or farmland, weed seeds were evenly sown in pots (840 mm length × 560 mm width × 360 mm height) with high density to simulate about 100% weed cover. The barnyard grass (*Echinochloa crusgalli* L.), sedge grass (*Cyperus rotundus* L.), weedy rice (*Oryza sativa* L.), and sprangletop (*Leptochloa chinensis* L.), which all grow in farmland and uncultivated land around Nanjing city, were selected as weeds. These weed seeds were mixed randomly based on weight, at a ratio of 1:1:1:1, and then uniformly sown in each test pot (840 mm length × 560 mm width × 360 mm height) with the same amount of seeds, ten pots of each treatment were prepared. After 40 d of weed growth, the density and the average cover of the weeds were calculated from ten pots per treatment. According to the results, the average densities of barnyard grass, weedy rice, sedge, and sprangletop per pot were approximately 550 ± 60 plants/m^2^ (dominant weeds), 60 ± 10 plants/m^2^, 120 ± 30 plants/m^2^, and 450 ± 50 plant/m^2^ under the farmland with weed competition, and were approximately 460 ± 50 plants/m^2^ (dominant weeds), 50 ± 12 plants/m^2^, 110 ± 40 plants/m^2^, and 420 ± 60 plant/m^2^ uncultivated land with weed competition, respectively, and the initial weed cover was 100%.

### Experimental Design

This experiment was conducted from May to October 2016 in a glass greenhouse at the Nanjing Environmental Science Institute of the Ministry of Ecology and Environment under low insect pressure free of target-insect. The experiment was laid in a randomized block design with four growing conditions based on different combinations of land use and weeds to compare the fitness of exogenous Cry1C* gene to parental rice. Combined treatments included the following: farmland without weed competition (F – NW, the control treatment), uncultivated land without weed competition (U – NW, semi-natural individual treatment), farmland with weed competition (F – W, semi-natural individual treatment) and uncultivated land with weed competition conditions (U – W, natural combination treatment). Considering the rice seeds will germinate along with weed seeds under wild uncultivated land, the rice seeds were simultaneously and directly sown (eight seeds per hole) into above–mentioned pots at a density of 200 mm × 200 mm along with weed seeds. Rice pot experiments without weeds were used as controls. Other seedlings were pulled out to ensure one plant in each hole after emergence. Rice plant height was significantly lower than that of weeds (8-10 cm) at this point, which was affected by weed competition. Finally, 12 plants were evenly distributed in each pot, ten pots of each treatment were prepared. A total of 80 pots were prepared for the two rice lines in eight treatments (i.e. 960 plants) randomly placed in the greenhouse. In addition, except for the higher density of heteromorphic sedge during seedling, tillering, and heading stages, sedge basically disappeared at the grain filling stage, while other weeds developed over the entire life cycle of the rice plants. Overall, weed coverage rate increased initially up to 100% before grain filling but then decreased to 80% at maturity. Weeds were intensively controlled over the whole growth stage under F – NW and U-NW treatments. No insecticide was applied to the rice plants under any of the four treatments, and all other experimental materials were incinerated and inactivated after the test was completed.

### Investigation of Insect in the Experiments

Forty pots were randomly selected to estimate the non-target (such as planthopper) or target insect (*Scirpophaga incertulas, Chilo suppresses, Sesamia inferens and Cnaphalocrocis medinalis*) number in glass greenhouse. The investigation sites per pot were set up in the four corners and the middle position of T1C-19 and MH63, respectively. Data was collected at jointing and heading stages of the rice plants.

### The Expression of Exogenous Protein Was Determined by the Enzyme-linked Immunosorbent Assay

On July 20 (at tillering), August 5 (at jointing), September 5 (at heading), September 25 (at filling) and October 25 (at maturing), leaves and stems of five plants were sampled and mixed into one duplicate sample in each plot and five such duplicate pots from each treatment were stored at −80°C for determination of Cry1C* protein content by the enzyme-linked immunosorbent assay kit (EnviroLogix Inc, Portland, ME, USA). The detailed analytical procedure was carried out according to Fu et al.^18^

### Detection of Vegetative and Reproductive Growth Indices of Rice

Four plants randomly selected in the middle of each plot were used for measurement of plant height, tiller number, and SPAD value for the flag leaf at tillering, heading, grain filling and maturity stages, five duplicated pots from each treatment were processed. The aboveground parts of 10 plants randomly selected each treatment were oven-dried at 80°C and weighed for biomass determination at maturity.

At maturing stage, selecting the 20 rice plants that measure the vegetative growth indices to analyze the following reproductive growth indices under four growth conditions: (1) effective number of panicles (2) spike length; (3) spike weight; (4) total grain number per plant; (5) filled grain number per plant; (6) filled grain weight per plant; (7) 1000-grain weight; and (8) seed setting rate. The detailed determination method is referred to Fu et al.^18^

### Data Collection and Analysis

The expression of Cry1Ab/c protein in the same tissues and at the same growth stage were compared and analyzed using the independent variable t-test under different growth conditions. Spatiotemporal dynamic changes of expression of the Cry1Ab/c exogenous protein in different plant tissues at different growth stages were analyzed by Duncan´s multiple comparison method under the same growth condition.

Relative differences between T1C-19 and MH63 rice in terms of vegetative and reproductive growth indices were compared with an independent *t*-test. Three-way ANOVA was applied to estimate the effect of two soil conditions, two rice lines, and three or four growth stages on vegetative performance. Two-way ANOVA was applied to estimate the effect of two soil conditions and two rice lines on reproductive performance.

According to the method of Song et al.,^19^ the independent sample *t*-test was used to test whether the fitness value (T1C *vs* MH63) was significantly different from 1.0. All aforementioned statistical analyses were computed using SPSS v.16.0 for Windows (IBM Corp, Armonk, NY, USA).

## Results

### Insects in the Experiments

There were only a few non-target insects such as Arachnida, Coccinellidae and Locusta migratoria manilensis in the greenhouse. We did not find planthopper, and observe dead heart and leaf rolling of rice plants caused by rice stem borers (Scirpophaga incertulas, Chilo suppresses and Sesamia inferens) and rice leaf borers (Cnaphalocrocis medinalis) because there is (Table S1) no farmland and crops within 5 km of this independent glass greenhouse. Thus, the pot experiments described herein were effectively low insect pressure, without the target insect.

## *Expression of Exogenous* Cry1C* *Protein*

**Figure 1.**
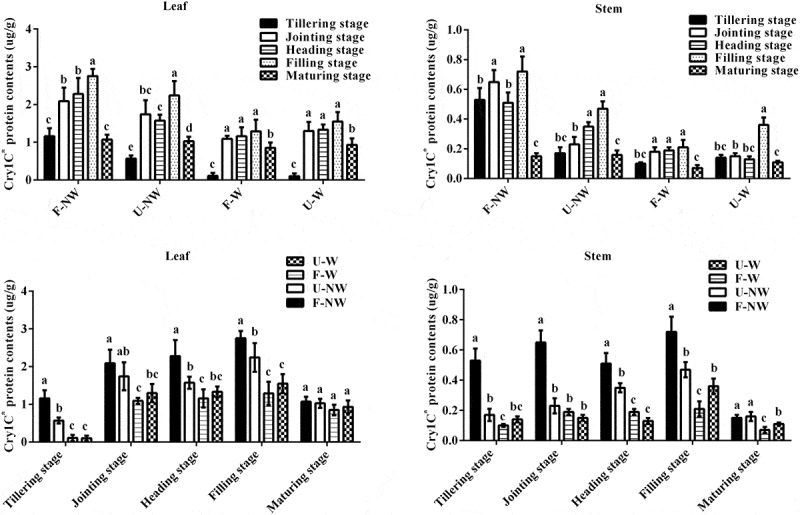
Cry1C* protein expression in leaves and stems under four simulated growing conditions during different growth stages in T1C-19. Lowercase letters indicate significant differences among the five growth stages grown under the same conditions or grown under four conditions at the same growth stage of T1C-19 rice according to Duncan’s multiple range test (n = 5, *P* < .05)

According to Figure 1, the expression of Cry1C* protein in transgenic rice T1C-19 leaves and stems showed spatiotemporal expression specificity during different growth stages under the same growth conditions. It increased first and then decreased along with the growth stage, remained the highest expression at the filling stage, and decreased significantly at the maturity stage.

Under different growth conditions, Cry1C* protein expression in T1C-19 leaves and stems showed some differences in the same growth stage, including the expressions of exogenous Cry1C* protein under U–NW, F–W and U–W conditions were significantly lower than that in the control condition, F–NW.

In conclusions, according to the analysis results of three-way ANOVA, the expressions of Cry1C* protein were significantly affected by different growth conditions, different growth stages, different tissues and their interaction (Table S2).

### Vegetative Growth Indices

#### Plant Height

**Figure 2.**
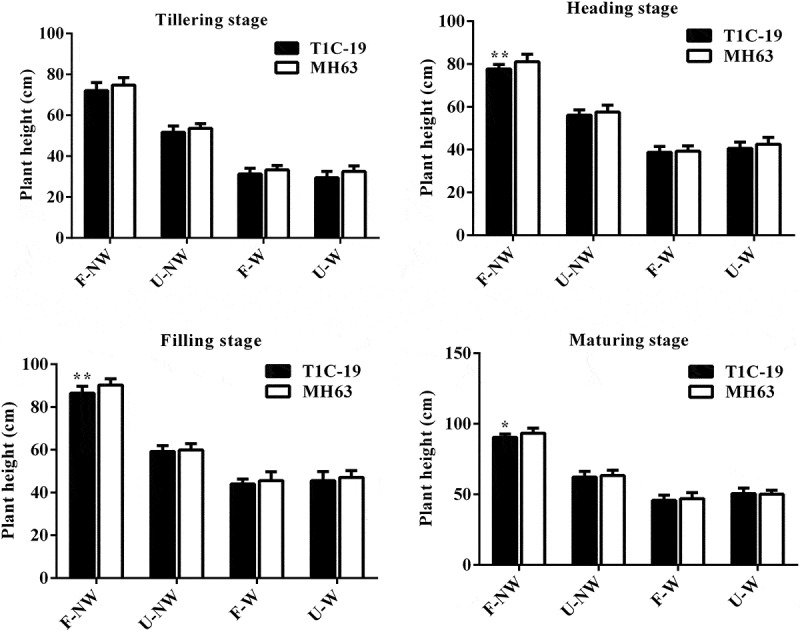
**Plant height (mean ± SEM) of T1C-19 and MH63 rice cultivated under four growing conditions combining land use and weeds**. Values for T1C-19 rice with * and ** are significantly different from those for MH63 according to *t*-test (*P* < .05) and (*P* < .01), respectively (n = 20)

The plant height of T1C-19 rice and MH63 rice at the same growth stage presented some differences under different growth conditions. The plant heights of the two rice lines under U–NW, F–W and U–W conditions were significantly lower than in the control condition, F–NW (*P* < .01, Figure 2).

Under the same growth conditions, the change trends of plant height in T1C-19 rice and MH63 rice during the whole growth period were similar, both gradually increased with the growth process. Under F–NW conditions, the plant heights of T1C-19 rice were significantly lower than that of MH63 rice by about 3%–4% at heading stage, filling stage and maturing stage (*P* < .01). Under U–NW, F–W and U–W conditions, there was no significant difference in plant height between T1C-19 rice and MH63 rice during the whole growth stage.

Furthermore, according to the analysis results of three-way ANOVA, exogenous gene, different growth conditions, different growth stages and the interaction between growth conditions and growth stages all had significant effects on rice plant height. However, the interaction between exogenous genes and growth conditions, exogenous genes and growth stage, and exogenous gene, growth conditions and growth stages had not significant effect on rice height (Table S3).

#### Tiller Number

**Figure 3.**
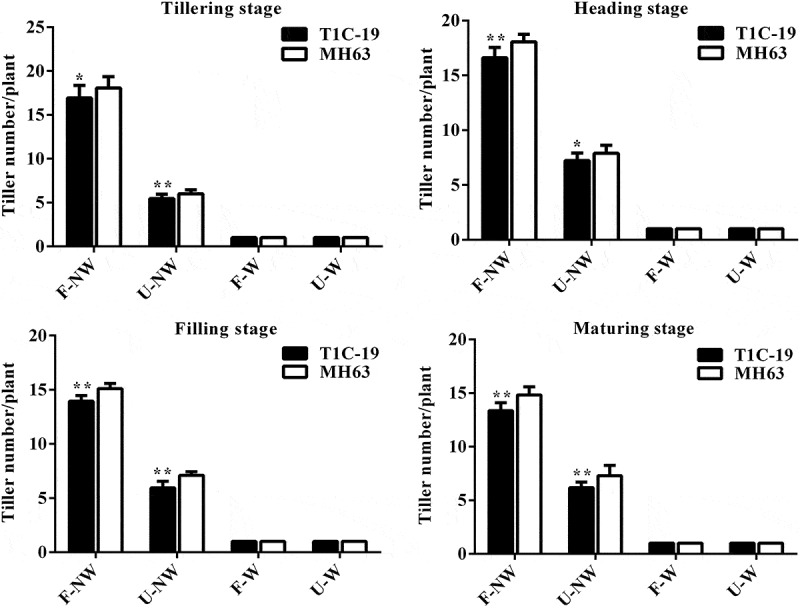
**Tilling numbers per plant (Means ± SEM) of T1C-19 and MH63 rice cultivated under four growing conditions combining land use and weeds**. Values for T1C-19 rice with * and ** are significantly different from those for MH63 according to *t*-test (*P* < .05) and (*P* < .01), respectively (n = 20)

The tiller numbers of T1C-19 rice and MH63 rice at the same growth stage presented some differences under different growth conditions. The tiller numbers of two rice lines under U–NW, F–W and U–W conditions were significantly lower than that under the control condition, F–NW (*P* < .01, Figure 3).

Under the same growth conditions, the change trends of tiller number in T1C-19 rice and MH63 rice during the whole growth period were similar, both increased first and then decreased with the growth stage. The tiller numbers of T1C-19 rice were significantly lower than that of MH63 rice by about 6–10% during four stages under F–NW condition (*P* < .01). Similarly, the tiller numbers of T1C-19 rice were significantly lower than that of MH63 rice by about 8–16% (*P* < .01) during four stages under U-NW condition, and the magnitude of significant difference in tiller number between T1C-19 rice and MH63 rice under this U–NW condition was bigger than that of F–NW condition. There was no tiller between T1C–19 rice and MH63 rice during four stages under F–W and U–W conditions, (tiller number was “1.00”).

Furthermore, according to the analysis results of three-way ANOVA, exogenous gene, different growth conditions, different growth stages, the interaction between exogenous genes and growth conditions, the interaction between growth conditions and growth stages all significantly affected rice tiller number. However, the interaction between exogenous genes and growth stages and the interaction of exogenous genes, growth conditions and growth stages had not significant effect on rice tiller number (Table S3).

#### SPAD Value of Flag Leaf

**Figure 4.**
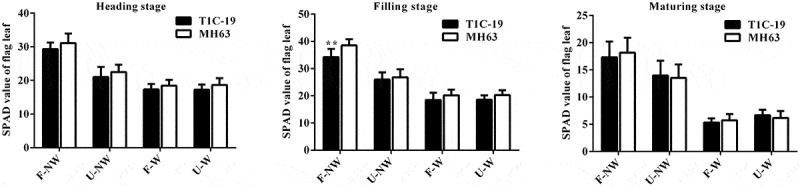
**SPAD values (Means ± SEM) of T1C-19 and MH63 rice cultivated under four growing conditions combining land use and weeds**. Values for T1C-19 rice with ** are significantly different from those for MH63 according to *t*-test (*P* < .01, n = 20)

The SPAD values of flag leaves of T1C-19 rice and MH63 rice showed some differences at the same growth stage under different growth conditions. The SPAD values of flag leaves of the two rice lines under U–NW, F–W and U–W conditions were significantly lower than that under the control condition, F–NW (*P* < .01, Figure 4).

Under F–NW condition, the SPAD value of flag leaf in T1C-19 rice was only significantly lower than that of MH63 rice by 11% at the filling stage (*P* < .01). Under U–NW, F–W and U–W conditions, SPAD values of flag leaf in T1C-19 did not have significantly difference from those of MH63 rice at heading stage, filling stage and maturing stage.

Furthermore, according to the analysis results of three-way ANOVA, exogenous genes, different growth conditions, different growth stages, the interaction between exogenous genes and growth conditions, the interaction between growth conditions and growth stages, the interaction between exogenous genes and growth stage all significantly affected rice SPAD value. However, the interaction of exogenous genes, growth conditions and growth stages had not significant effect on SPAD value of flag leaf (Table S3).

#### Biomass

**Figure 5.**
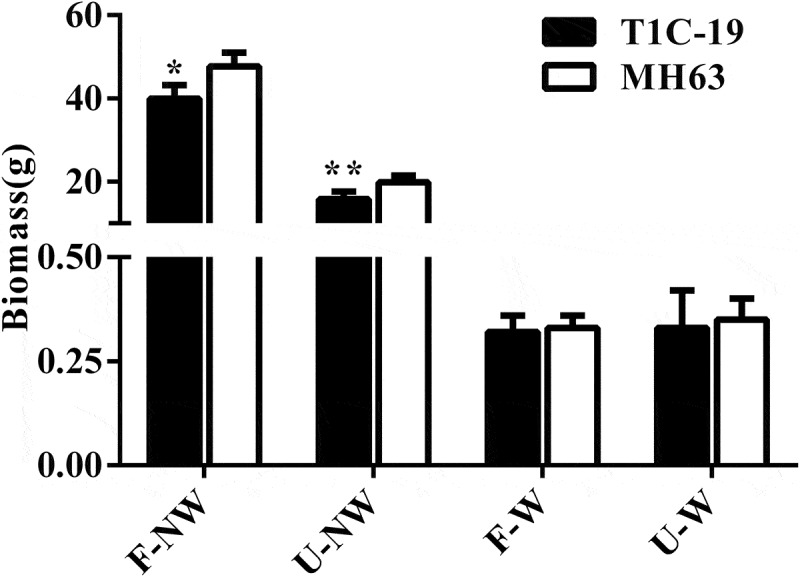
**Biomass (Means ± SEM) of T1C-19 and MH63 rice cultivated under four growing conditions combining land use and weeds**. Values for T1C-19 rice with * and ** are significantly different from those for MH63 according to *t-*test (*P* < .05) and (*P* < .01), respectively (n = 10)

The biomass of T1C-19 rice and MH63 rice presented some differences under different growth conditions, the biomass of the two rice lines under U–NW, F–W and U–W conditions was significantly lower than that under the control condition, F–NW at maturing stage (*P* < .01, Figure 5).

The biomass of T1C-19 rice was significantly lower than that of MH63 rice by about 16% under F–NW condition (*P* < .01). Similarly, the biomass of T1C–19 rice was significantly lower than that of MH63 rice by about 20% under U–NW condition (*P* < .01). There were no significant differences of biomass between T1C-19 rice and MH63 rice under F–W and U–W conditions.

Furthermore, according to the analysis results of three-way ANOVA, it was found that exogenous genes, growth conditions and their interaction significantly affected rice biomass (Table S3).

### Reproductive Growth Indices

Overall, the reproductive indices of T1C-19 rice and MH63 rice showed different fitness performance under different growth conditions: two lines under U–NW, F–W and U–W conditions were significantly lower than those of the control condition, F–NW (*P* < .01, Table 2).

**Table 2.** Reproductive indices of T1C-19 and MH63 rice genotypes cultivated under four growing conditions combining land use and weeds

Reproductive indices	F-NW		U-NW		F-W		U-W	
	T1C-19	MH63	T1C-19	MH63	T1C-19	MH63	T1C-19	MH63
Effective panicle number/plant	9.33 ± 0.58**	12.67 ± 1.25	4.18 ± 0.39**	5.80 ± 0.60	1.00 ± 0.00	1.00 ± 0.00	1.00 ± 0.00	1.00 ± 0.00
Panicle length (cm)	25.03 ± 0.31	24.22 ± 1.27	20.58 ± 1.47	20.39 ± 1.81	10.99 ± 0.76*	9.02 ± 1.21	9.93 ± 1.19*	11.09 ± 1.80
Panicle weight (g)	27.42 ± 1.17**	33.62 ± 1.87	7.59 ± 0.54**	13.52 ± 1.05	0.19 ± 0.03	0.20 ± 0.02	0.29 ± 0.08	0.28 ± 0.04
Filled grain number/plant	878.00 ± 57.87**	1068.17 ± 82.71	268.67 ± 26.90**	393.83 ± 33.88	7.00 ± 1.29	6.87 ± 0.78	9.37 ± 1.49	9.64 ± 1.61
Grain weight/plant (g)	20.39 ± 1.72**	23.43 ± 1.27	6.71 ± 0.67**	9.85 ± 0.85	0.18 ± 0.04	0.17 ± 0.02	0.23 ± 0.04	0.24 ± 0.04
Grain number/plant	1223.67 ± 57.90**	1409.00 ± 48.74	309.80 ± 23.79**	438.50 ± 32.71	10.66 ± 0.94	10.85 ± 1.55	13.50 ± 1.65	13.64 ± 2.46
Thousand grain weight (g)	26.04 ± 0.55	26.82 ± 0.40	26.07 ± 0.50	26.65 ± 0.71	25.30 ± 0.73	25.63 ± 0.36	25.01 ± 0.58	25.60 ± 0.77
Seed-setting rate(%)	72.00 ± 7.00	75.74 ± 4.07	86.66 ± 3.64	89.74 ± 2.38	65.27 ± 6.24	61.33 ± 4.21	69.28 ± 5.25	71.16 ± 6.00

Means ± SEM followed by T1C-19 rice with * and ** are significantly different from MH63 rice according to *t*-test (*P* < 0.05) and (*P* < 0.01), respectively (n = 20).

The effective panicle number, panicle weight, total grain number per plant, filled grain number per plant, filled grain weight per plant were significantly lower than MH63 rice about 25.36%, 18.68%, 12.94%, 17.42% and 13.52%, respectively, under F–NW condition (*P* < .01). Similarly, the effective panicle number, panicle weight, total grain number per plant, filled grain number per plant, filled grain weight per plant were significantly lower than MH63 rice about 26.67%, 43.06%, 31.53%, 31.52% and 28.56%, respectively, under U–NW condition (*P* < .01). There were no significant differences of other important reproductive indices between T1C-19 rice and MH63 rice except for panicle length under F–W and U–W conditions.

Furthermore, according to the results of two-way ANOVA analysis, growth conditions, exogenous gene and their interaction had significant effects on most yield indices except for spike length and seed setting rate (*P* < .05, Table S4).

### Fitness

Overall, according to Table 3, the T1C-19 rice showed a significantly higher fitness cost than MH63 rice in terms of vegetative growth fitness, including tiller number and biomass under F–NW and U–NW conditions (*P* < .05). Conversely, there was no significant difference in vegetative growth fitness between T1C-19 and MH63 under F–W or U–W conditions.

**Table 3.** Fitness for vegetative and reproductive indices of T1C-19 *vs*. MH63 rice grown under four growth conditions combining land use and weeds

			F-NW	U-NW	F-W	U-W
Vegetative indices	Fitness	Height	0.95*	0.98	0.97	0.97
		Tiller number	0.88*	0.92*	1.00	1.00
		Biomass	0.84*	0.80*	0.94	0.96
		SPAD	0.93*	0.98	0.94	0.98
	Composite fitness		0.90*	0.92*	0.96	0.98
Reproductive indices	Fitness	Effective panicle number per plant	0.74*	0.72*	1.00	1.00
		Panicle length (cm)	1.03	1.00	1.22*	0.89*
		Panicle weight (g)	0.82*	0.56*	0.95	1.03
		Filled grain number per plant	0.82*	0.68*	1.07	0.97
		Filled grain weight per plant (g)	0.87*	0.68*	1.06	0.96
		Grain number per plant	0.87*	0.71*	0.98	0.99
		Thousand grain weight (g)	0.97	0.98	0.99	0.98
		Seed-setting rate (%)	0.95	0.96	1.06	0.97
	Composite fitness		0.88*	0.78*	1.04	0.97

Fitness ratio was defined as agronomic traits of T1C-19 *vs*. MH63 rice; total fitness was defined as the average value of all fitness for vegetative traits or reproductive traits. * indicates fitness significantly more than or less than 1.00 according to *t*-test (*P* < 0.05).

As for reproductive growth fitness, overall T1C-19 showed a significantly higher fitness cost than MH63 under F–NW and U–NW conditions, for example, effective panicle number per plant, panicle weight, total grain number per plant, grain number per plant and grain weight per plant (*P* < .05). Furthermore, there were no significant differences between T1C-19 and MH63 under F–W and U–W conditions in fitness regarding other important reproductive indices, except for panicle length.

## Discussion

Wild relatives of cultivated rice, such as common wild rice and weedy rice, among others, are distributed across all planting areas of cultivated rice in China. If insect-resistant transgenic rice varieties are cultivated commercially on a large scale in the future, there might be growing concerns regarding their spreading these varieties into natural ecosystems through volunteer plants or gene flow will result in potential ecological risks. Therefore, it is important to assess the fitness benefits or costs of insect-resistant transgenic rice germplasms under different natural environments for scientific management and assessment of the ecological consequences of its release on a large scale. However, there are currently very few reports on the fitness of the insect-resistant transgenic rice T1C-19 in natural ecosystems.

## Expression of the Exogenous Protein Cry1C* under Four Growing Conditions

Knowledge of the expression of exogenous proteins in natural ecosystems is a precondition for evaluating the fitness of exogenous proteins on recipient crop species. This study indicates that the expression of the exogenous Cry1C* protein in leaves and stems of T1C-19 rice showed spatiotemporal dynamic changes with different degrees over the crop cycle under the four different growing conditions, combining land use and weed competition. Previous studies have found that the expression of the Bt protein in insect–resistant transgenic rice, cotton, and maize leaves showed spatiotemporal dynamic changes in farmlands.^20–23^ In addition, the external environment had a significant effect on the expression of exogenous Cry1C* protein in T1C-19 rice; thus, individual and combined stress conditions U–NW, F–W or U–W, significantly inhibited the expression of Cry1C* protein in T1C-19 rice, compared to plants under control (F–NW) conditions. Similar studies have found that the expression of the exogenous *Bt* protein in insect-resistant transgenic rice under salinity, P deficiency, K deficiency, and other adverse conditions, were significantly lower than under no-stress control conditions.^18,24,25^ The negative correlation between *Bt* protein expression and stress has also been reported in insect–resistant transgenic cotton and maize.^26–30^ Taken together, these findings indicated that environmental stress limited the expression of exogenous proteins in insect-resistant transgenic crops; however, these proteins were still expressed in varying degrees. This may affect vegetative and reproductive growth fitness of transgenic rice T1C-19 under stress conditions.

Although the expression of exogenous genes driven by a constitutive promoter, such as CaMV 35S, several studies have shown that they produce spatiotemporal effects, and are also significantly affected by external environmental conditions,^24,25,31^ and plant growth and development.^32,33^ A possible explanation is that the expressions of exogenous genes is usually regulated at a post-transcriptional or translational level, and that transgene silencing is environmentally and developmentally regulated. In this study, we showed that weed competition can significantly affect the growth of the transgenic rice plants T1C-19 such as by competition for light and nutrients from soil, thus affecting the expression of the Cry1C* in T1C-19. This appears as a new environmental factor could affect the expression of exogenous protein through affecting the transgenic plants growth and development.

### Effects of Growing Conditions on Vegetative and Reproductive Fitness of Transgenic Rice

From an ecological perspective, different natural stress conditions have varying effects on the fitness of vegetative and reproductive growth of transgenic rice, which may lead to diverse ecological consequences. The results reported herein showed that the vegetative growth indices of T1C-19 and MH63 rice, such as plant height, tiller number and biomass, as well as reproductive growth indices, such as grain number per plant, grain weight per plant and others under individual and combined stress conditions U–NW, F–W and U–W, were significantly lower than those under the F–NW control condition. These results are similar to those reported previously, which showed that vegetative growth indices of insect-resistant transgenic rice, cotton, and maize under salt or drought stress were significantly lower than under no-stress growing conditions.^12,18,26,34,35^

**Figure 6.**
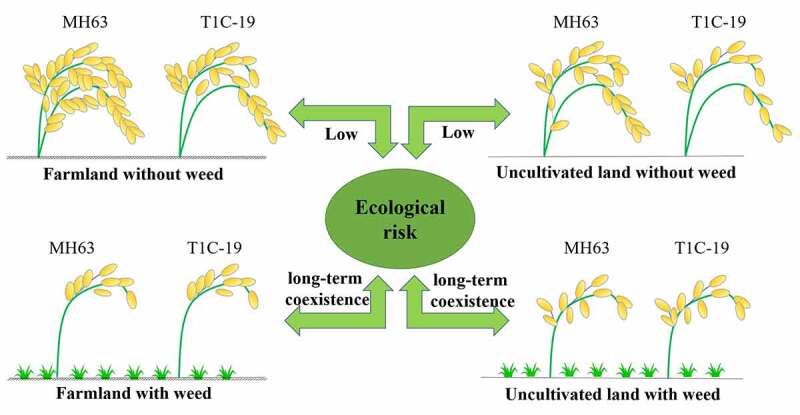
A low ecological risk of T1C-19 compared to MH63 under farmland and uncultivated land without weed, and ecological risk of their long-term coexistence under farmland and uncultivated land with weed

Additionally, tiller number, biomass and other important vegetative growth indices or grain number per plant, grain weight per plant and other important reproductive growth indices were significantly lower in TIC-19 than in the parental rice line, MH63, under F–NW and U–NW conditions, indicating that the transgene conferred a significant fitness cost. A possible explanation is that in the absence of biotic stress conditions, including target insect or weed pressures, the expression of insect-resistant exogenous protein may lead to significant fitness cost for the parental rice line. Our findings are consistent with those of Wang et al.,^17^ who found that transgenic Cry1C* rice showed lower grain yield than the parental-line MH63 in the field under natural low insect pressure, mainly due to reduced grain-filling percentage. Similarly, Jiang et al.^36^ found that transgenic Cry1C* rice had lower grain yield than the parental-line MH63 under nitrogen stress or no-stress conditions (control) due to reduced grain filling percentage. Other studies have shown that biomass, grain weight, and mean seed-setting rate of transgenic Cry1C* rice were significantly lower than those of the parental-line MH63 under phosphorus-deficient, potassium-deficient, or drought stress conditions.^24,25,37^ In contrast, there were no significant differences between T1C-19 and MH63 in tiller number, biomass, and other important vegetative or reproductive growth indices under F–W and U–W conditions. A possible explanation is that under biotic stress conditions, such as weed pressure, the expression of exogenous protein is too low to cause significant fitness to the parental line. This was consistent with previous reports that the expression of exogenous proteins was positively correlated with phenotypic differences in crops.^38,39^ Taken together, the findings of the present study showed that if transgenic Cry1C* rice T1C-19 escaped from the cultivation system into F–NW and U–NW ecosystems, it would likely not succeed in establishing a population and spread into these two ecosystems to any greater extent than its parental-line MH63, thereby posing a very low ecological risk. However, if T1C-19 rice escaped and entered into a F–W or the U–W ecosystem, it may co–grow with MH63, and thus lead to the successful establishment of a T1C-19 rice population that may persist for a long time in these ecosystems, thereby resulting in potential ecological risks (Figure 6).

Here, except for foreign gene Cry1C*, the genetic background of transgenic rice T1C-19 is the same as that of its parental rice MH63, and therefore these phenotypic differences between T1C-19 and MH63 under the same environment are most likely attributed to foreign gene Cry1C*. To determine the range of the differences induced by foreign gene Cry1C*, we will introduce the comparison between parental plants MH63 and other wild rice relatives harboring different genetic backgrounds, compared with the differences between parental plants MH63 and transgenic plants T1C-19 using transcriptome, proteome and metabolome methods in the future. Furthermore, more numbers of other transgenic and non-transgenic varieties will be used for risk assessment in the natural ecosystem to strengthen our results.

## Conclusions

In conclusion, T1C-19 showed weaker vegetative and reproductive growth abilities than MH63 in farmland or uncultivated land without weed competition; consequently, any ecological risk due to its persistence in the environment was assessed as a low risk. Conversely, under farmland or uncultivated land with weed competition, no significant differences were detected in the vegetative or reproductive growth abilities between T1C-19 and MH63. This finding implies a significant potential ecological risk due to the joint growth of the two genotypes in a shared natural ecosystem. Therefore, we predicted that the fitness and ecological risk of transgenic crops in the different stressful environments may not merely relate to the expression of the exogenous protein, but will also be affected by the environment. Our study clearly distinguished the environmental effects combining land use and weed competition that may give rise to a high potential ecological risk associated with the scope of the transgene through volunteer plants or gene flow into the interphase areas between paddy farmland and surrounding natural ecosystems upon large-scale cultivation of transgenic rice. Currently, as transgenic rice has not been approved for field planting according to the Chinese law, in this study, we only selected two natural factors (soil collected from uncultivated field and weed competition to simulate the natural ecosystem in the greenhouse) for predicting the possible ecological risks associated with planting the transgenic rice-line T1C-19. However, in reality, there are several additional factors decide the natural ecosystem. Therefore, future research should assess the ecological risks of planting the transgenic rice-line T1C-19 under real wild natural environments such as grassland and shrub habitats, that have low target insect pressure. Although no studies have shown that the tissue culture process for the construction transgenic rice T1C-19 and location effect of exogenous Cry1C* insertion cause above-mentioned unintended effects, these factors should be considered in the design of future experiments.

## Supplementary Material

Supplemental Material

## Data Availability

The data sets supporting the conclusions of this article are included within the article and its supplementary information
