# Supplementary material for: Fitness of Insect-resistant transgenic rice T1C-19 under four growing conditions combining land use and weed competition
Source: GM Crops Food. 2021 Apr 21;12(1):328–41. doi: 10.1080/21645698.2021.1914290 (PMC8086590; doi:10.1080/21645698.2021.1914290)
Supplement: Supplemental Material [file KGMC_A_1914290_SM0679.docx]

**Supplementary Information**

**Table S1**

Non-target and target insect number per pot at jointing and heading stages in the greenhouse

| **Respondents** | **Jointing stage** | | **Heading stage** | |
| --- | --- | --- | --- | --- |
|  | **Non-target insect** | **Target insect** | **Non-target insect** | **Target**  **insect** |
| Arachnida | 5.4±2.1/pot | -- | 5.5±2.4/pot | -- |
| Coccinellidae | 8.1±2.6/ pot | -- | 9.3±2.5/ pot | -- |
| *Manilensis* (*Meyen*) | 6.1±1.4/pot | -- | 7.2±2.1/pot | -- |
| Planthopper | 0.0±0.0/pot |  | 0.0±0.0/pot | -- |
| *S. incertulas* | -- | 0.0±0.0/pot | -- | 0.0±0.0/pot |
| *C. suppressalis* | -- | 0.0±0.0/pot | -- | 0.0±0.0/pot |
| *C. medinalis* | -- | 0.0±0.0/pot | -- | 0.0±0.0/pot |

**Table S2**

Three-way ANOVA of the effects of growing condition, growth stage, plant tissue, and interactions among them, on Cry1C^*^ protein expression in transgenic T1C-19 rice

| Three-way ANOVA | Cry1C^*^ protein (μg/g) | |  | |
| --- | --- | --- | --- | --- |
|  | df | F | *p* |  |
| Gc | 3 | 72.15 | 0.00 |  |
| Gs | 4 | 67.05 | 0.00 |  |
| T | 1 | 826.96 | 0.00 |  |
| Gc × Gs | 12 | 3.25 | 0.00 |  |
| Gc × T | 3 | 14.44 | 0.00 |  |
| Gs × T | 4 | 38.30 | 0.00 |  |
| Gc × Gs × T | 12 | 1.16 | NS |  |

Gc: Growth condition, Gs: Growth stage, T: Tissue. P < 0.05 indicates significant difference; NS indicates no significant difference

**Table S3**

Three-way ANOVA of the effects of plant growing conditions, rice line, growth stage and the interactions among them, on vegetative growth indices

| Three-way ANOVA | Plant height (cm) | | | Tiller number | | | SPAD value of flag leave | | | Biomass (g) | | |
| --- | --- | --- | --- | --- | --- | --- | --- | --- | --- | --- | --- | --- |
|  | df | F | P | df | F | P | df | F | p | df | F | P |
| Gc | 3 | 3,786.78 | 0.00 | 3 | 1531.78 | 0.00 | 3 | 678.69 | 0.00 | 3 | 1159.83 | 0.00 |
| Rl | 1 | 29.42 | 0.00 | 1 | 65.78 | 0.00 | 1 | 18.94 | 0.00 | 1 | 24.51 | 0.00 |
| Gs | 3 | 420.33 | 0.00 | 3 | 3.82 | 0.00 | 2 | 1362.21 | 0.00 | - | - | - |
| Gc×Rl | 3 | 1.45 | NS | 3 | 41.68 | 0.00 | 3 | 2.91 | 0.03 | 3 | 9.58 | 0.00 |
| Gc × Gs | 9 | 9.95 | 0.00 | 9 | 11.62 | 0.00 | 6 | 16.07 | 0.00 | - | - | - |
| Rl× Gs | 3 | 0.67 | NS | 3 | 1.57 | NS | 2 | 6.14 | 0.00 | - | - | - |
| G×Rl× Gs | 9 | 0.41 | NS | 9 | 1.36 | NS | 6 | 0.69 | NS | - | - | - |

Gc: Growth condition, Rl: Rice lines, Gs: Growth stages. p < 0.05 indicates significant difference; NS indicates no significant difference.

**Table S4**

Two-way ANOVA of the effects of plant growing condition, rice line and their interaction on reproductive indices

| Reproductive indices | Gc | | | Rl | | | Gc × Rl | | |
| --- | --- | --- | --- | --- | --- | --- | --- | --- | --- |
|  | df | F | P | df | F | P | df | F | P |
| Effective panicle number/plant | 3 | 1,721.69 | 0.00 | 1 | 127.81 | 0.00 | 3 | 46.23 | 0.00 |
| Panicle length (cm) | 3 | 368.41 | 0.00 | 1 | 1.43 | NS | 3 | 3.08 | 0.03 |
| Panicle weight (g) | 3 | 2,927.27 | 0.00 | 1 | 127.91 | 0.00 | 3 | 43.88 | 0.00 |
| Grain number/plant | 3 | 5,931.22 | 0.00 | 1 | 101.06 | 0.00 | 3 | 35.63 | 0.00 |
| Filled grain number/plant | 3 | 1,879.51 | 0.00 | 1 | 57.68 | 0.00 | 3 | 20.89 | 0.00 |
| Filled grain weight/plant (g) | 3 | 2,158.30 | 0.00 | 1 | 50.31 | 0.00 | 3 | 16.94 | 0.00 |
| Thousand grain weight (g) | 3 | 6.41 | 0.00 |  | 2.21 | NS |  | 1.89 | NS |
| Seed-setting rate (%) | 3 | 12.20 | 0.00 | 1 | 0.66 | NS | 3 | 1.45 | NS |

Gc: Growth condition, Rl: Rice lines, Gs: Growth stages. P < 0.05 indicated significant difference; NS indicated no significant difference.
